# Adipose-derived stem cells-seeded bladder acellular matrix graft-silk fibroin enhances bladder reconstruction in a rat model

**DOI:** 10.18632/oncotarget.21211

**Published:** 2017-09-23

**Authors:** Dongdong Xiao, Qiong Wang, Hao Yan, Xiangguo Lv, Yang Zhao, Zhe Zhou, Ming Zhang, Qian Sun, Kang Sun, Wei Li, Mujun Lu

**Affiliations:** ^1^ Department of Urology and Andrology, Ren Ji Hospital, School of Medicine, Shanghai Jiao Tong University, Shanghai 200001, China; ^2^ Department of Urology, The Sun Yat-sen Memorial Hospital, Sun Yat-sen University, Guangzhou 510120, China; ^3^ The State Key Lab of Metal Matrix Composites, School of Materials Science and Engineering, Shanghai Jiao Tong University, Shanghai 200240, China

**Keywords:** bladder augmentation, adipose stem cell, silk fibroin, bladder acellular matrix graft, angiogenesis

## Abstract

The unfavourable clinical outcomes of host cell-seeded scaffolds for bladder augmentation warrant improved bioactive biomaterials. This study aimed to examine the feasibility of adipose-derived stem cells (ASCs)-seeded bilayer bladder acellular matrix graft (BAMG)-silk fibroin (SF) scaffold in enhancing bladder reconstruction. Sprague Dawley rats were randomly divided into three groups: the BAMG-SF-ASCs group, the acellular BAMG-SF group and the cystotomy group. The BAMG-SF-ASCs group was sampled at 2, 4 and 12 weeks, and compared with the other groups at 12 weeks. In the BAMG-SF-ASCs group, the normal bladder contour was reformed similar to that in the cystotomy group, with abundant urothelium and smooth muscle regeneration, as well as a suitable scaffold degradation speed, and trivial fibrosis and inflammation. The ASCs seeded in BAMG-SF were maintained in the regenerated region during the 12-week experimental period and significantly enhanced the vessel density, nerve regeneration and bladder function compared with acellular BAMG-SF. In addition, the BAMG-SF-ASCs group presented elevated levels of SDF-1α, VEGF and their receptors, with an obvious increase in ERK 1/2 phosphorylation. BAMG-SF is a promising biomaterial for ASCs seeding to facilitate bladder augmentation and demonstrated an enhanced angiogenic potential possibly related to the SDF-1α/CXCR4 pathway via ERK 1/2 activation.

## INTRODUCTION

Bladder augmentation is necessitated in various conditions, such as neurogenic bladder, bladder exstrophy, congenital bladder abnormalities, interstitial cystitis, and trauma, as well as cystectomy for bladder cancer [1]. Conventional enterocystoplasty has prevailed for decades as the gold standard for bladder augmentation, despite the subsequent high possibility of various complications, including urinary tract infections, urolithiasis, bowel segment perforation and secondary malignancy, as well as donor site morbidity and electrolyte imbalance [2].

In the context of the eminent need for new alternatives to enterocystoplasty, tissue-engineered bladder augmentation holds great promise for overcoming the related obstacles and meeting this need. Contemporary bladder tissue engineering strategies lack the ability to regenerate bladder smooth muscle and vasculature, and promote peripheral nerve tissue growth when using autologous cell populations. Polyglycolic-acid (PGA) seeded with autologous smooth muscle and urothelial cells was a pioneer in the treatment of myelomeningocele patients with hampered bladder compliance [3]. However, the favourable outcomes of this pilot study were later threatened by the results of the phase II study, which showed unsatisfactory bladder compliance after long-term follow-up with serious adverse events [4], as well as unbeneficial bladder compliance improvements and insufficient smooth muscle regeneration resulting from the simple application of an acellular collagen matrix in bladder augmentation for bladder exstrophy patients [5].

In an effort to solve these problems, there has been an increasing trend of replacing autologous cells with stem cells in bladder augmentation with synchronous improvements in scaffold properties. In addition to invasive procedures for harvesting from the bladder, smooth muscle and urothelial cells are subject to senescence during *in vitro* cultivation [6, 7]. Moreover, the functional status of autologous cells isolated from diseased bladder tissue is highly suspect, potentially compromising their performance *in vivo*. The use of stem cells circumvents the disadvantages of using autologous cells, and stem cells have exhibited a recognized potential in regenerative medicine [8]. Compared with embryonic stem cells and bone marrow-derived stem cells, adipose-derived stem cells (ASCs) are associated with fewer ethical considerations and are more easily accessible via liposuction or abdominoplasty [9]. ASCs are mesenchymal stromal cells found in the perivascular space of adipose tissue, with the advantage of abundance and easy access compared with other stem cell types [10]. Adipose tissue contains 100-1,000 times more pluripotent cells on a per-cubic centimetre basis than bone marrow [11]. In addition to their multi-lineage differentiation potency, ASCs are notable for their abundant secretome that mediated angiogenesis, wound healing, tissue regeneration, and immune cell reactions [12, 13]. It has been indicated that ASCs can secrete angiopoietin 1, vascular endothelial growth factor (VEGF), nerve growth factor, brain-derived neurotropic factor and glial cell-derived neurotropic factor to promote angiogenesis and innervation [14], which promote bladder reconstruction.

Despite the extensive applications of various stem cells seeded on natural or synthetic scaffolds in attempts to facilitate bladder reconstruction, numerous obstacles remain to be overcome. In our previous study, ASCs seeded on bladder acellular matrix graft (BAMG) in combination with intraperitoneal incubation vanished at 4 weeks post-implantation in a rat model, as determined by histological examination [15]. PGA was reported to be associated with excessive lymphocyte infiltration and slow degradation at 8 weeks [16]. ASCs-seeded tissue-engineered prepuce scaffolds only enlarged bladder capacity by approximately 1.3-fold at 90 days, with an unsatisfactory number of CD31^+^ vessels in a rat model [17].

A bilayer BAMG-silk fibroin (SF) scaffold was shown in our previous study to effectively facilitate bladder regeneration in a time-dependent manner [18]. In this study, we hypothesized that the bilayer BAMG-SF scaffold could convey ASCs to further promote bladder reconstruction. BAMG is an acellular collagen-based biomatrix derived from bladder and accommodates bladder function by exhibiting excellent waterproofness against urine and extraordinary mechanical properties [19]. However, it was also found to have a low porosity and an inability to sustain ASCs for the entire bladder regenerative period. Derived from *Bombyx mori* silkworm cocoons, SF exhibits broad processing plasticity, including a three-dimensional (3D) porous configuration, as well as good biocompatibility, high structural strength and elasticity, and tuneable biodegradability [20]. However, the simple SF structure may increase the risk of urinary calculus and urinary leakage [21]. The combined advantages of BAMG and SF render the bilayer BAMG-SF scaffold potentially suitable for ASCs seeding to repair bladder defects. This application was comprehensively examined in this study in terms of morphology, histology, serum biochemistry and functionality. In addition, the possible roles of ASCs were investigated, as well as their contribution to bladder-scaffold integration and tissue regeneration.

## RESULTS

### The interaction between the ASCs and bilayer BAMG-SF scaffold

**Figure 1:**
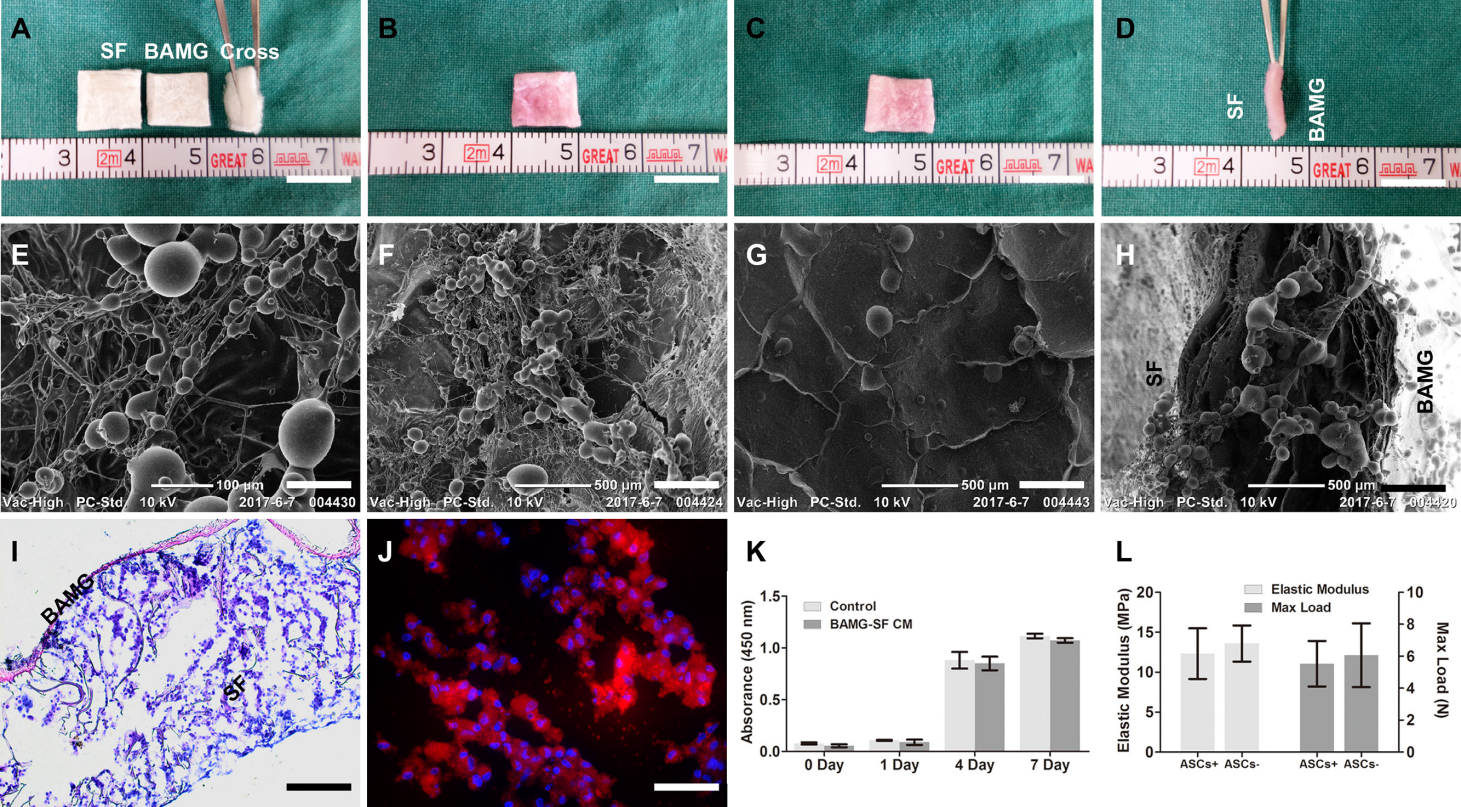
ASCs were seeded in cytocompatible BAMG-SF in large quantities without drastically altering its mechanical properties Representative photographs of the acellular BAMG-SF scaffold (BAMG, SF and cross-sectional views) **(A)** and the ASCs-seeded BAMG-SF scaffold from the porous SF side **(B)**, the waterproof BAMG side **(C)** and the bilayer cross-sectional view **(D)**. Representative SEM images displaying ASCs inside the SF layer **(E)**, the microstructure of porous SF layer **(F)** and BAMG layer **(G)** and the cross-sectional view **(H)**. **(I)** ASCs spread across the whole SF layer after a 3-day incubation *in vitro*, as shown by H&E stain. **(J)** ASCs were present as separate cells or cell clusters inside the SF layer under immunofluorescence, as shown by CM-DiI (red) labelling and DAPI (blue) counterstaining. **(K)** The BAMG-SF-conditioned media did not alter the proliferation of ASCs significantly within 1 week *in vitro*, suggesting the good cytocompatibility of BAMG-SF. **(L)** Incubation with ASCs for 3 days had no significant influence on the elastic modulus or maximum load of BAMG-SF. Gross view scale bar = 1 cm; SEM scale bar = 100 μm **(E)**; SEM scale bar = 500 μm **(F-H)**; H&E scale bar = 100 μm; immunofluorescence scale bar = 50 μm. Data are expressed as the mean ± standard deviation. Cell proliferation experiments and mechanical tests were performed in triplicate.

The BAMG-SF scaffold (1 cm×1 cm) applied in this study had a bilayer configuration consisting of a waterproof BAMG layer and a porous SF layer with a thickness of approximate 1.5 mm (Figure 1A). The gross morphology of the BAMG and SF did not change drastically after being seeded with ASCs, as determined by BAMG, SF and cross-sectional views (Figure 1B, 1C, 1D). In particular, the porous SF layer did not collapse after a three-day culture with ASCs *in vitro* (Figure 1B). Many ACSs were dispersed along the interconnected microfilaments inside the porous SF layer, as observed by scanning electron microscopy (SEM, Figure 1E). Most of the ACSs adhered to the interconnected filaments of the SF layer were separate or exhibited fused cell membranes, while few cells remained floating on the surface (Figure 1F). A small quantity of ASCs could also be found on the BAMG and cross-sectional sides (Figure 1G, 1H). ASCs were disseminated throughout the entire porous SF layer upon the relatively dense BAMG layer, as shown by haematoxylin-eosin (H&E) stain (Figure 1I), and these cells were viewed as separate cells or clusters in the fluorescent images of CM-DiI-stained samples counterstained by 4′,6-diamidino-2-phenyllindole (DAPI) (Figure 1J). The good cytocompatibility of BAMG-SF was in line with the discovery that the ASCs proliferated rapidly within 7 days of culture in either BAMG-SF-conditioned medium or control medium, without significant differences (P>0.05 at each time point, Figure 1K). Moreover, the superior combination of the structural strength and elasticity of BAMG-SF was not significantly altered by ASCs seeding for 3 days *in vitro*, with an elastic modulus of 12.32 ± 3.17 MPa and a max load of 5.53 ± 1.42 N. The collective data suggested that cytocompatible BAMG-SF could be seeded abundantly with ASCs without significant alterations to its morphology or mechanical properties.

### Body weight, bladder calculus, haematology and serum biochemistry analyses

The rats in all three groups gained comparable body weight during the desirable survival period (Table 1). The BAMG-SF induced similar bladder calculus whether seeded with ASCs or not, while little bladder calculus was detected in the cystotomy group. All three groups showed a sign of transient acute inflammation, i.e., elevated white blood cell counts at 2 weeks, which returned to a normal level at 4 and remained normal through 12 weeks (Table 2). There were no significant deviations from the physiological references of serum alanine transferase (ALT), aspartate transferase (AST), blood urea nitrogen (BUN), creatinine (Cr), potassium, sodium or chloride in any group. These findings suggested that ASCs-seeded BAMG-SF caused no liver or renal dysfunction, unremitting systemic toxicity or metabolic disturbance in the experimental animals.

**Table 1:** Body weight change and bladder calculus of SD rats at different timepoints

Times (Weeks)	Weight pre-op (g)	Weight post-op (g)	Weight change (g)	Calculus number (n)	Calculus weight (μg)
2 Weeks					
BAMG-SF-ASCs	200.5 ± 9.0	290.6 ± 9.5	90.0 ± 1.6	0	0
BAMG-SF	205.4 ± 9.4	282.9 ± 17.7	77.5 ± 8.6	0	0
Cystotomy	203.5 ± 5.8	287.1 ± 9.8	83.6 ± 14.5	0	0
4 Weeks					
BAMG-SF-ASCs	210.5 ± 5.8	334.4 ± 7.4	123.9 ± 13.1	1.7 ± 0.6	3.5 ± 0.9
BAMG-SF	209.6 ± 13.0	331.1 ± 11.0	121.6 ± 23.9	2.3 ± 0.6	4.9 ± 1.7
Cystotomy	209.3 ± 10.7	333.2 ± 13.2	123.9 ± 6.8	0	0
12 Weeks					
BAMG-SF-ASCs	196.1 ± 4.0	448.5 ± 14.5	252.4 ± 15.9	3.7 ± 1.5	7.2 ± 2.8
BAMG-SF	202.8 ± 13.4	455.1 ± 16.8	252.3 ± 13.3	4.3 ± 2.1	8.4 ± 3.6
Cystotomy	208.1 ± 14.0	447.8 ± 5.2	239.8 ± 17.7	0	0

Notes: Data are expressed as the mean ± standard deviation.

**Table 2:** Blood cell counts and serum biochemistry of SD rats at different timepoints

Time (Weeks)	WBC (10^9/L)	RBC (10^12/L)	ALT (U/L)	AST (U/L)	BUN (mmol/L)	Cr (μmol/L)	K^+^ (mmol/L)	Na^+^ (mmol/L)	Cl^-^ (mmol/L)
2 Weeks									
BAMG-SF-ASCs	**10.4 ± 1.2**	5.8 ± 0.6	49.6 ± 5.9	126.1 ± 32.2	6.4 ± 0.8	51.3 ± 7.2	5.2 ± 0.7	140.5 ± 3.1	104.3 ± 3.3
BAMG-SF	**10.2 ± 0.8**	5.6 ± 0.7	54.5 ± 4.5	120.0 ± 25.6	6.6 ± 0.6	48.9 ± 7.8	5.2 ± 0.8	137.7 ± 1.7	106.0 ± 2.9
Cystotomy	**10.7 ± 0.8**	6.1 ± 0.5	48.2 ± 5.8	119.6 ± 31.6	6.2 ± 1.4	52.3 ± 6.0	5.1 ± 0.5	142.9 ± 2.3	105.6 ± 2.6
4 Weeks									
BAMG-SF-ASCs	6.0 ± 0.8	6.4 ± 0.4	48.8 ± 4.8	104.6 ± 27.1	6.0 ± 0.7	54.1 ± 4.7	5.3 ± 0.5	140.3 ± 2.2	103.8 ± 2.2
BAMG-SF	5.9 ± 0.5	6.1 ± 0.7	50.0 ± 6.6	127.6 ± 27.6	5.8 ± 1.0	51.6 ± 6.6	4.8 ± 0.3	138.6 ± 2.8	103.9 ± 3.3
Cystotomy	6.1 ± 0.6	6.2 ± 0.8	48.9 ± 6.6	109.3 ± 33.6	6.2 ± 1.2	49.1 ± 6.5	4.9 ± 0.6	137.9 ± 1.8	104.6 ± 2.6
12 Weeks									
BAMG-SF-ASCs	5.8 ± 0.4	6.0 ± 0.6	45.2 ± 5.2	113.3 ± 30.8	5.7 ± 1.0	50.6 ± 5.1	5.1 ± 0.8	139.8 ± 3.4	105.9 ± 3.4
BAMG-SF	6.0 ± 0.5	6.2 ± 0.4	51.1 ± 6.8	130.1 ± 12.7	5.4 ± 1.2	48.0 ± 3.8	4.7 ± 0.7	139.5 ± 1.9	106.3 ± 3.0
Cystotomy	5.6 ± 0.2	5.9 ± 0.6	47.0 ± 7.0	116.0 ± 25.7	5.2 ± 1.2	51.0 ± 6.5	5.2 ± 0.4	140.7 ± 3.9	101.9 ± 1.8

Notes: Data are expressed as the mean ± standard deviation. All experiments were performed in triplicate. Data in bold indicate a significant difference compared with the other timepoints in the same group (P<0.05).

### Morphological recovery of bladder gross appearance and retrograde cystography

**Figure 2:**
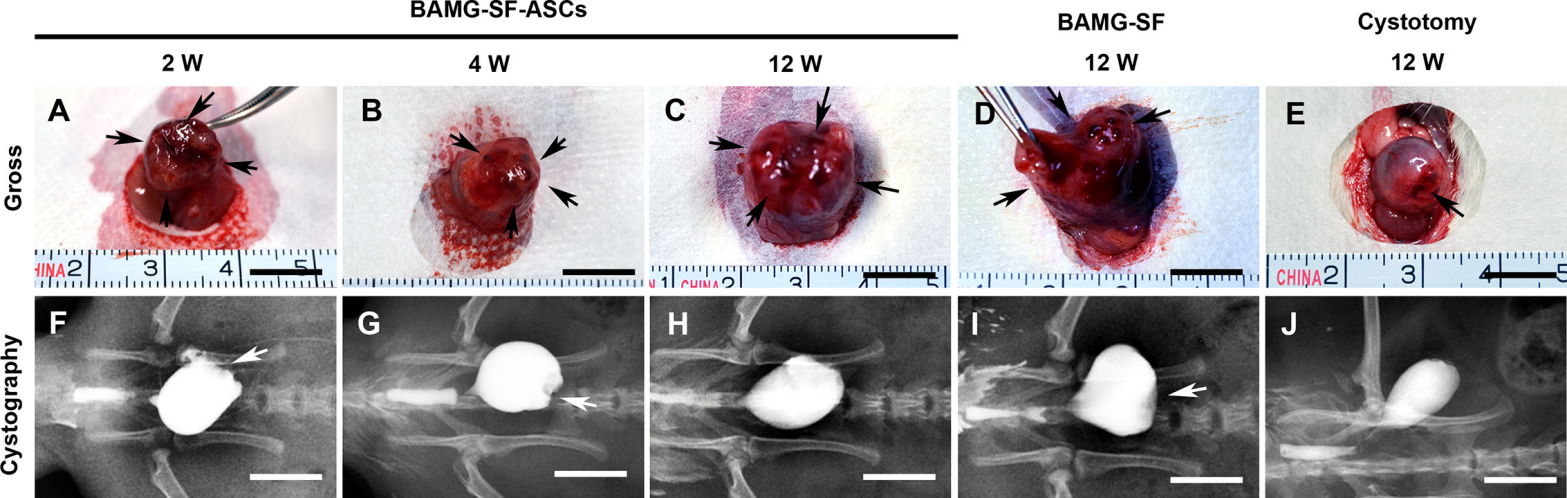
ASCs-seeded BAMG-SF promoted morphological restoration of bladder defect Representative gross photographs of regenerated bladder tissue *in vivo* with ASCs-seeded BAMG-SF at 2 **(A)**, 4 **(B)** and 12 **(C)** weeks and acellular BAMG-SF **(D)** and cystotomy **(E)** at 12 weeks. Representative retrograde cystography photographs of bladder augmented by ASCs-seeded BAMG-SF at 2 **(F)**, 4 **(G)** and 12 **(H)** weeks and acellular BAMG-SF **(I)** and cystotomy **(J)** at 12 weeks. Black arrows mark the remaining sutures and regenerated tissue present *in vivo* within the original implantation sites supported by scaffolds. White arrows note depression of the regenerated area under retrograde cystography. Scale bar = 1 cm.

Trivial adhesions were found between the bladder and adjacent omentum and intestine. After carefully dissecting the adhesions, negligible scar formation and graft shrinkage were observed in the regenerated bladder tissue distinctly margined by the aforementioned non-absorbable sutures (Figure 2). The ASCs-seeded BAMG-SF entirely supported the original defect in a flat appearance, which could be palpated at 2 weeks (Figure 2A). Thereafter, the implantation gradually developed from an irregular surface at 4 weeks (Figure 2B) into a relatively oval shape (Figure 2C) similar to the control group (Figure 2E) at 12 weeks. In contrast, the acellular BAMG-SF still exhibited a pouch-like irregular shape at 12 weeks (Figure 2D). The gradual transformations of the gross appearance were also revealed by retrograde cystography. The ASCs-seeded BAMG-SF facilitated presentation of the regenerated area as a straight notch at 2 weeks (Figure 2F), which gradually developed into a small depression at 4 weeks (Figure 2G) and finally disappeared at 12 weeks (Figure 2H). Unlike the flat pouch-like shape of the bladder in the acellular BAMG-SF group (Figure 2I), the BAMG-SF-ASCs group presented an oval-shaped bladder similar to that of the control group (Figure 2J). The results indicated that BAMG-SF-ASCs scaffold gradually promoted the morphological restoration of the regenerated bladder.

### Histological restoration with suitable SF degradation rate, mild fibrosis and inflammation

**Figure 3:**
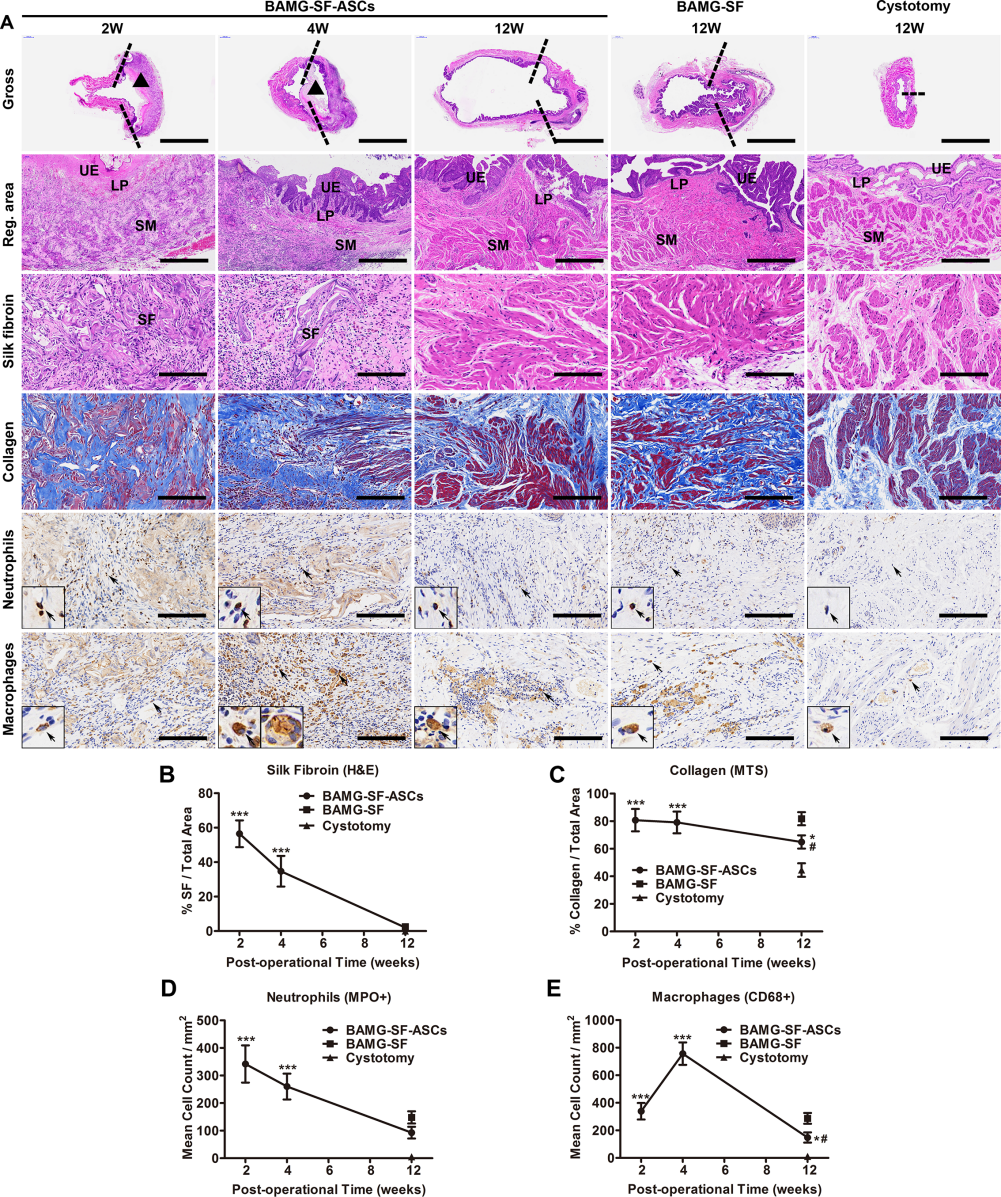
ASCs-seeded BAMG-SF reformed bladder wall gradually with matched biodegradation and mild inflammation **(A)** Representative images of entire bladder longitudinal sections, bladder regenerated areas, SF (homogeneous pink areas or fragments), collagen (blue), MPO^+^ neutrophils and CD68^+^ macrophages. Histomorphometric quantitative comparison of **(B)** silk fibroin, **(C)** collagen, **(D)** MPO^+^ neutrophils, and **(E)** CD68^+^ macrophages. Reconstructed areas are labelled by dashed lines. The BAMG-SF scaffold is marked by black triangles. Typical MPO^+^ neutrophils and CD68^+^ macrophages are denoted by black arrows and displayed in magnified inserts. Gross view: 40×, scale bar = 5 mm; reg. area: 100×, scale bar = 200 μm; SF, collagen, neutrophils and macrophages: 200×, scale bar = 100 μm. Data are expressed as the mean ± standard deviation. UE: urothelium; LP: lamina propria; SM: smooth muscle; SF: silk fibroin. *P<0.05, ***P<0.001 versus the cystotomy group; #P<0.05 versus the BAMG-SF group.

To explore the histological changes of the aforementioned morphological evolvements, corresponding histometric analyses were performed and demonstrated the major role of the BAMG-SF-ASCs scaffold at each stage of bladder regeneration. Supporting the initial bladder defect with infiltrating connective tissue and inflammatory cells in the SF layer, the BAMG-SF-ASCs scaffold was aligned with a thin layer of urothelium on the BAMG side and exhibited trivial smooth muscle regeneration at 2 weeks. Gradually, increasing proportions of smooth muscle bundles with multi-layered urothelium accumulated at 4 and 12 weeks (Figure 3A). The SF layer degraded in accordance with its replacement by host tissue *in vivo* from 2 weeks and vanished at 12 weeks (Figure 3A, 3B). Extensive fragmentation of the porous compartment of BAMG-SF was noted within the regenerated lamina propria at 2 weeks. The SF degraded gradually *in vivo* with qualitatively higher levels of residual scaffold remnants present within the central bladder wall compared with the radial peripheral areas, suggesting that degradation of the bilayer scaffold proceeded in a radial fashion from the border of the original implantation site towards the centre region as host tissue integration proceeded. Masson’s trichrome stain (MTS) revealed the wane of collagen and wax of smooth muscle during the regeneration period (Figure 3A). In the BAMG-SF-ASCs group, sparse smooth muscle fibres replaced collagen fibres progressively from 2 weeks and developed into highly defined, well-organized muscle fascicles interspersed with a level of collagen fibres (64.88 ± 4.79%) that was greater than that in the control group (44.54 ± 4.90%, P<0.05) but significantly lower than that in the BAMG-SF group (81.80 ± 4.75%, P<0.05) (Figure 3C). Myeloperoxidase^+^ (MPO^+^) neutrophils aggregated at the implantation site at 2 and 4 weeks as a sign of transient acute inflammation and dissipated rapidly at 12 weeks in the BAMG-SF-ASCs group, similar to the BAMG-SF and control groups (Figure 3A, 3D). CD68^+^ macrophages began to gather around the SF fragments at 2 weeks and increased in quantity by approximately one-fold at 4 weeks (Figure 3A, 3E), when some macrophages fused together into a multi-nuclear form with intracellular SF remnants. There were still a few clusters of CD68^+^ macrophages in the BAMG-SF-ASCs group compared with the control group (P<0.05), but these clusters were less severe than those in the BAMG-SF group (Figure 3E). The collective results indicated that ASCs seeded in BAMG-SF facilitated bladder histological restoration with a suitable scaffold degradation speed, and trivial fibrosis and inflammation.

### Immunofluorescence photometric analyses of bladder wall component regeneration

**Figure 4:**
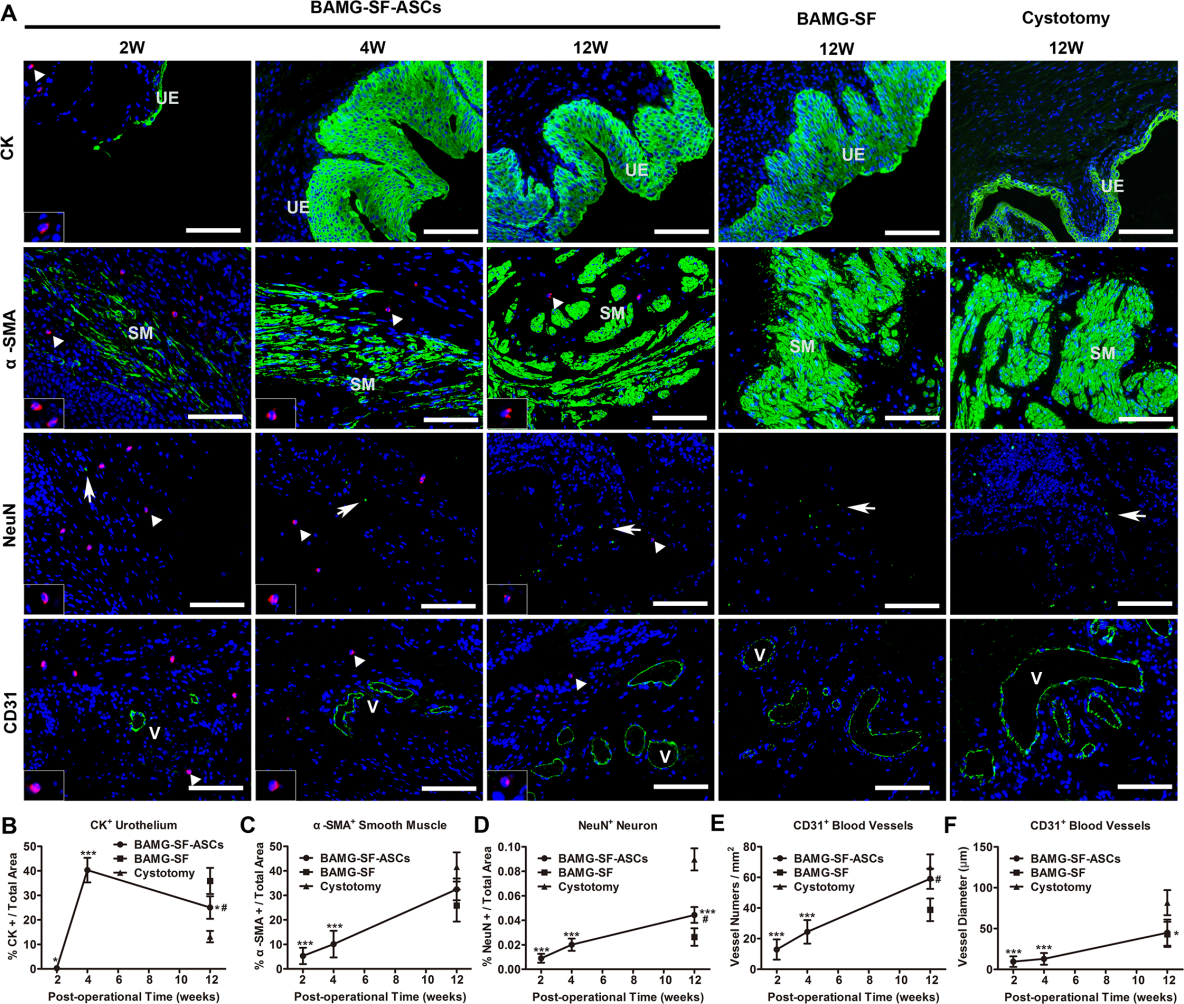
Immunofluorescence photometric analyses of respective bladder wall components **(A)** Representative immunofluorescence images of the urothelium marker CK, the smooth contractile muscle markerα-SMA, the neuronal marker NeuN, and the blood vessel endothelial marker CD31. Positive respective marker expression is shown in green (labelled by FITC), and the nuclei are counterstained by DAPI (blue); 200×, scale bar = 100 μm. Histomorphometric quantitative comparison of **(B)** CK^+^ urothelium, **(C)** α-SMA^+^ smooth muscle bundles, **(D)** NeuN^+^ neuronal boutons, **(E)** the mean number per mm^2^ and **(F)** the mean diameter of CD31^+^ vessels. UE: urothelium; SM: smooth muscle. Typical CM-DiI-labelled ASCs (red) are denoted by white triangles and displayed in magnified inserts; these cells could be detected throughout the 12-week regeneration period. White arrows note NeuN^+^ neuron boutons. “V” marks CD31^+^ blood vessels. Data are expressed as the mean ± standard deviation. *P<0.05, ***P<0.001 versus the cystotomy group; #P<0.05 versus the BAMG-SF group.

The respective components of the regenerated bladder wall were analysed by immunofluorescence and photometric assessments (Figure 4A). CM-DiI-labelled ASCs could be traced during the entire experimental period and exhibited a gradually decreasing number and fluorescence intensity. A small proportion of discontinuous cytokeratin^+^ (CK^+^) urothelium was detected at 2 weeks, which developed to be hyperplastic at 4 weeks. At 12 weeks, the CK^+^ urothelium recovered to a multilayer structure with a proportion lesser than that of the BAMG-SF group (P<0.05) but still presented a sign of hyperplasia compared with the control group (P<0.05) (Figure 4B). α-smooth muscle actin^+^ (α-SMA^+^) smooth muscle regenerated from sparse smooth muscle fibres at 2 weeks to increasing smooth muscle bundles at 4 weeks and finally achieved a proportion similar to that of the control group at 12 weeks (P>0.05), showing no significant difference from the BAMG-SF group (P>0.05) (Figure 4C). Unlike the irregular structure of α-SMA^+^ smooth muscle in the BAMG-SF group, that of the BAMG-SF-ASCs group consisted of regular transverse and longitudinal orientations, which were similar to that of the cystotomy group. The NeuN^+^ neurons in the BAMG-SF-ASCs group regenerated slowly from 2 weeks and reached a higher proportion than that in the BAMG-SF group at 12 weeks (P<0.05), which was still significantly different from that in the control group (49.40% of the control group, P <0.001) (Figure 4D). The CD31^+^ vessel number of the BAMG-SF-ASCs group steadily grew and was restored to a greater level than that of the BAMG-SF group (P<0.05), which was similar to that of the control group (P>0.05) (Figure 4E). However, both the BAMG-SF-ASCs and BAMG-SF groups fostered similar CD31^+^ vessel diameters (P>0.05), which were less favourable than those of the control group (P<0.05) (Figure 4F). These results suggested that ASCs seeded in BAMG-SF scaffold promoted angiogenesis and innervation in the regenerated bladder tissue.

### Augmented bladder capacity with physiological compliance

**Figure 5:**
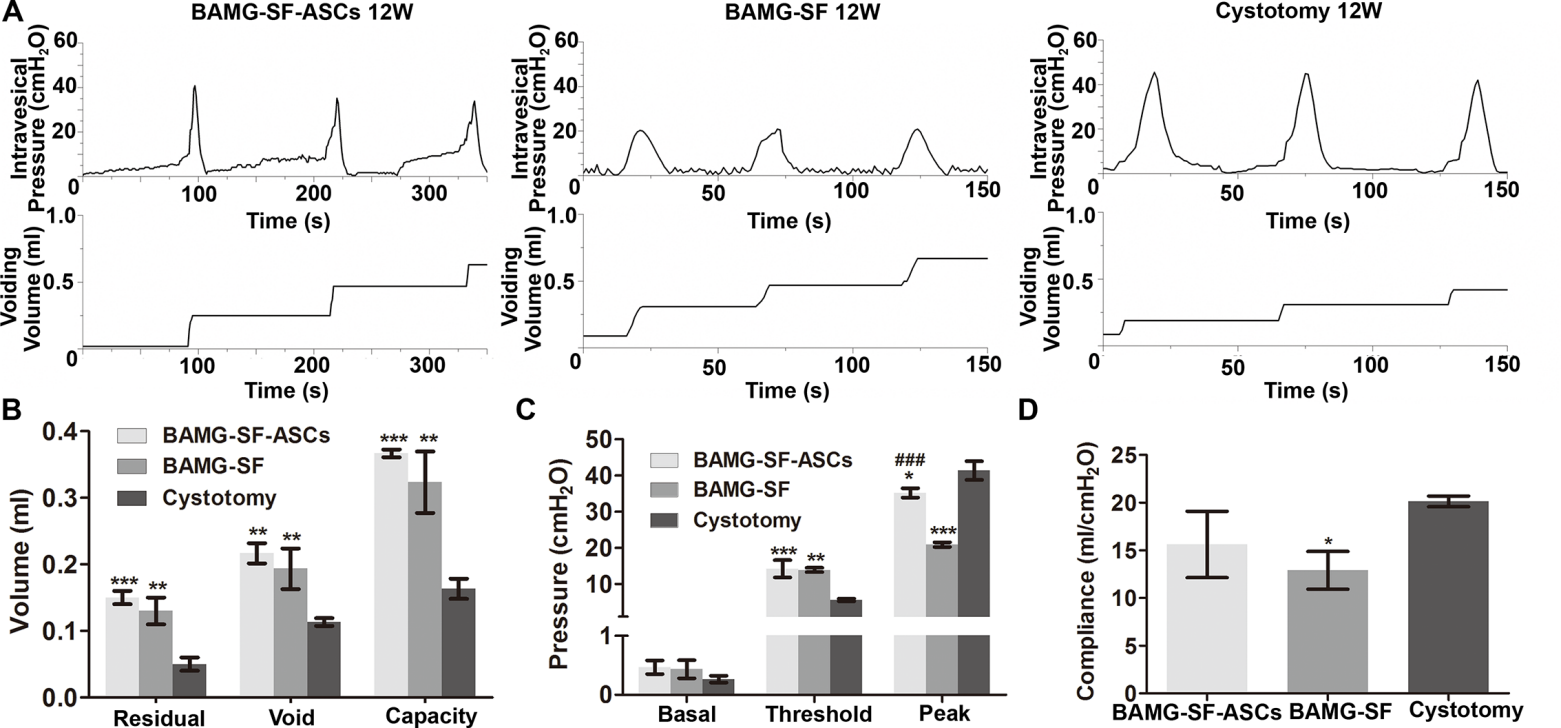
ASCs-seeded BAMG-SF enhanced bladder functional recovery with augmented capacity and physiological compliance **(A)** Representative cytograms of the groups augmented with ASCs-seeded BAMG-SF, acellular BAMG-SF and cystotomy at 12 weeks. **(B)** Residual volume, void volume, and bladder capacity at 12 weeks. **(C)** Bladder basal, threshold, and peak pressures at 12 weeks. **(D)** Bladder compliance at 12 weeks. All experiments were performed in triplicate. Data are expressed as the mean ± standard deviation. *P<0.05, **P<0.01, ***P<0.001 versus the cystotomy group; ###P<0.001 versus the BAMG-SF group.

Cystometric parameter measurements contributed to an understanding of the intricate relationship between architectural bladder tissue regeneration and physiological function. The BAMG-SF-ASCs group exhibited regular filling and voiding phases, as did the control group, without indications of obstruction or irritation (Figure 5A). The capacity of the BAMG-SF-ASCs group was augmented to 224.60% that of the control group (P<0.001), with increased residual volume and voiding volume (P<0.001 for residual volume; P<0.01 for voiding volume) (Figure 5B). The threshold pressure was elevated but the peak pressure was reduced slightly in the BAMG-SF-ASCs group compared with the control group (P<0.001 for threshold pressure; P<0.05 for peak pressure) (Figure 5C). While the BAMG-SF-ASCs group showed no significant difference from the control group in bladder compliance (BAMG-SF-ASCs: 15.61 ± 3.48 ml/cmH_2_O, control: 20.13 ± 0.57 ml/cmH_2_O, P>0.05), that of the BAMG-SF group (12.91 ± 1.98 ml/cmH_2_O) was lower than the control level (P<0.05) (Figure 5D). There were no significant differences in the urodynamic parameters between the BAMG-SF-ASCs and BAMG-SF groups, except for a drastic decline in peak pressure in the BAMG-SF group (P<0.001). These data indicated that ASCs seeded in BAMG-SF helped to restore bladder function with augmented capacity.

### ASCs activated stromal cell-derived factor 1α (SDF-1α)/ chemokine receptor 4 (CXCR4) pathway to enhance VEGF-mediated angiogenesis via extracellular signal-regulated kinases 1/2 (ERK 1/2) activation

**Figure 6:**
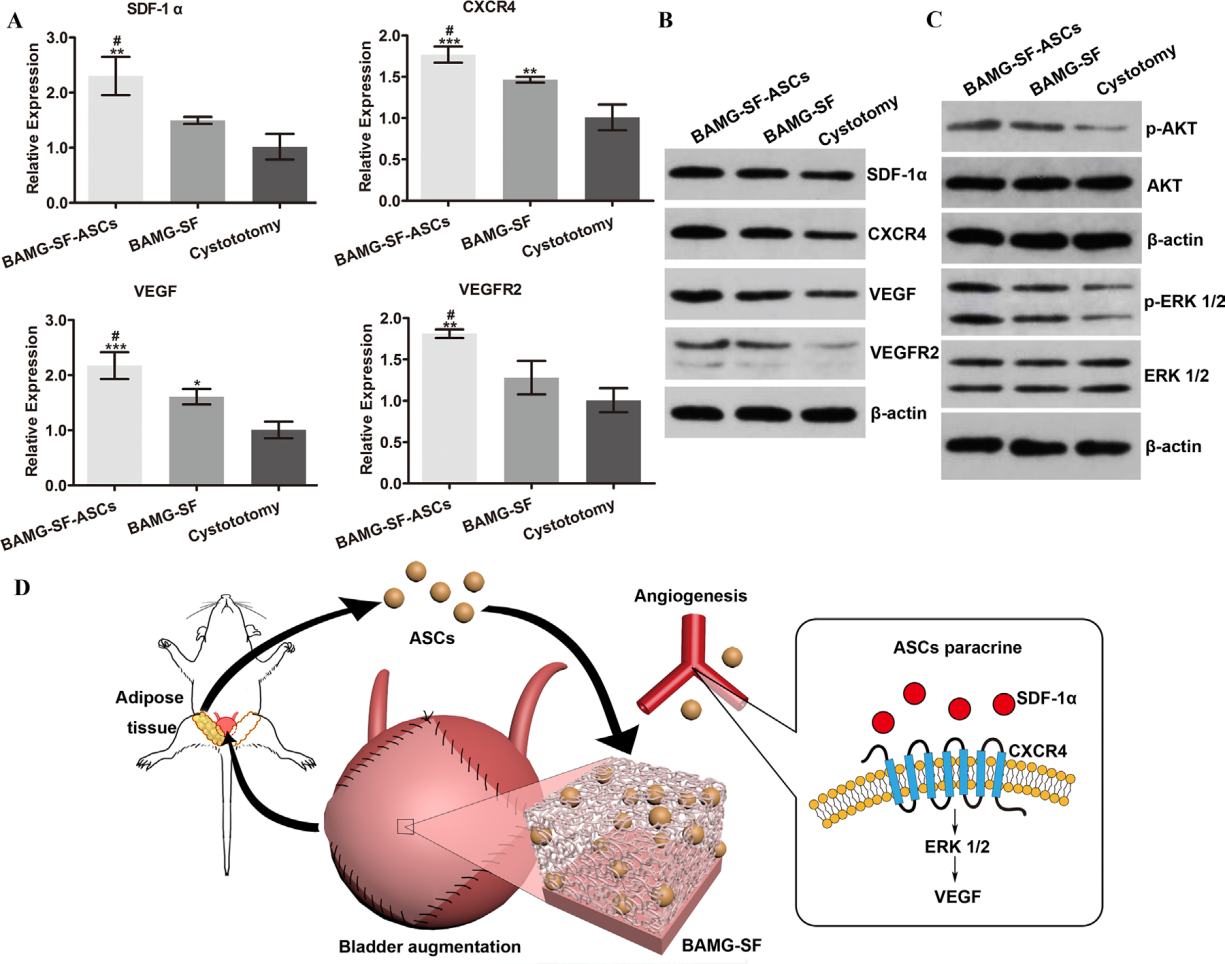
Enhanced angiogenic potential of ASCs via the SDF-1α/CXCR4 pathway and potentially involved mechanisms **(A)** The transcriptional levels of SDF-1α, CXCR4, VEGF and VEGR2 were significantly elevated in bladder augmented with ASCs-seeded BAMG-SF compared with bladder augmented with acellular BAMG-SF. **(B)** ASCs seeded in BAMG-SF enhanced SDF-1α, CXCR4, VEGF and VEGFR2 protein expression levels in regenerated bladder tissue. **(C)** The protein expression levels of AKT and ERK 1/2 were similar among all three groups. An obvious increase in phosphorylated ERK 1/2, but not phosphorylated AKT, was observed in the ASCs-seeded BAMG-SF group. **(D)** A schematic illustration of the study design and enhanced bladder regeneration facilitated by ASCs-seeded BAMG-SF. Data are expressed as the mean ± standard deviation. *P<0.05, **P<0.01, ***P<0.001 versus the cystotomy group; #P<0.05, versus the BAMG-SF group.

The transcriptional levels of SDF-1α and its receptor CXCR4 were significantly elevated in the BAMG-SF-ASCs group compared with the BAMG-SF group (P<0.05). Correspondingly, ASCs increased the transcription of VEGF and VEGR2, which promoted neovascularization (Figure 6A). The quantitative real-time polymer chain reaction (qRT-PCR) findings were corroborated by the Western bolt analysis, indicating increased protein expression levels of SDF-1α, CXCR4, VEGF and VEGF receptor 2 (VEGFR2, Figure 6B). The protein expression levels of protein kinase B (AKT) and ERK 1/2 were stable in all three groups. ERK 1/2 phosphorylation, but not AKT phosphorylation, was obviously facilitated by ASCs (Figure 6C). These results indicated that the phosphorylation of ERK 1/2 may be involved in the enhanced VEGF-mediated angiogenic potential of ASCs through the SDF-1α/CXCR4 pathway.

## DISCUSSION

Bladder tissue engineering is confronted by an urgent need to treat a variety of acquired and congenital bladder abnormalities as an alternative to conventional gastrointestinal bladder augmentation. Stem cell therapy has been shown to be a promising approach to enhance the regeneration of tissue-engineered bladder. However, faced with the limitations of current biomaterials and obstacles towards clinical applications, it is necessary to fabricate a novel suitable scaffold to convey stem cells *in vivo* and to investigate the underlying mechanisms of action.

The collective data obtained in this study demonstrate the efficacy and safety of the bilayer BAMG-SF scaffold seeded with ASCs in the morphological and functional restoration of bladder defects in a rat model of bladder augmentation by promoting angiogenesis with alleviated fibrosis and inflammation, which is associated with the SDF-1α/CXCR4 pathway via the activation of ERK 1/2 (Figure 6D).

The essential prerequisite of scaffolds for cell-based therapies lies in the maintenance of seeded cells during the regeneration process. There is a widespread consensus that properly degummed and sterilized silk products have good biocompatibility [22]. Moreover, the unique combination of elasticity, strength and biodegradation, along with good biocompatibility and low immunotoxicity, demonstrated by this study makes SF an attractive material for bladder tissue engineering. Our findings are consistent with those found after nanoparticle-labelled bone marrow stromal cells were maintained in the SF layer of a poly-L-lactide/SF scaffold in regenerated bladder tissue for 12 weeks [23].

Inflammation is a double-edged sword in the context of scaffold-assisted bladder reconstruction. While it plays a crucial role in scaffold degradation and chemotaxis, it also provokes fibrosis, scarring and implantation rejection, thereby deteriorating the regeneration process. This process is in part caused by extracellular matrix product accumulation in and around regenerating tissue creating an inhospitable growth environment. The host immune system has a significant impact on the degradation of 3D SF porous scaffolds, and the degradation of silk sponges has been shown to be mediated by macrophages [24]. Similarly, it was observed in this study that macrophages clustered around SF fragments and had intracellular SF debris at 4 weeks, suggesting that silk is not only biodegradable but also bioresorbable. In this study, the degradation time of BAMG-SF scaffold coincided with the bladder regeneration process, which is a critical requirement to maintain the mechanical properties and structural integrity of the engineered bladder during all stages of the regeneration process. In addition, SF layer degradation products could be safely metabolized and cleared from the host body. Unlike synthetic scaffolds, SF does not release acidic by-products or exhibited decreased mechanical properties very early during degradation, and thus retains its strength over a long time. Because of the low immunotoxicity of SF, bladder augmented by BAMG-SF-ASCs scaffold showed no signs of scarring or graft shrinkage. The mildly excessive collagen deposition could be alleviated by ASCs. The typical ratio of muscle to collagen in the bladder is approximately 1:1 across a variety of species. Drastic deviation from this ratio leads to bladder dysfunction. The muscle-to-collagen ratio is indicative of the level of bladder regeneration in areas engrafted with cell-seeded scaffolds. While this ratio reached approximately 20% at 4 and 10 weeks with the use of bone marrow stem cell-seeded elastomeric poly (1, 8-octanediol-co-citrate)-based thin films [25], it reached approximately 50% in our study. The rapid decline of neutrophils inside the regeneration area from 2 weeks is in accordance with the transient increase in white blood cells at 2 weeks, suggesting an acute inflammatory reaction without inflammatory dysregulation. The sequential chronic inflammation represented by CD68^+^ macrophages accelerated the degradation of SF fragments and was also recognized to be associated hyperplasia of the urothelial layer in our previous study [18]. Some studies have suggested that stem cells can attenuate tissue inflammatory responses [26]. However, ASCs were not observed to reduce the rate of calculus formation in this study. These findings demonstrate that the inflammation caused by scaffold materials provoked stone formation and warrants more effective anti-inflammatory approaches.

The ability to recreate physiologically relevant organ functionality following substantial insult is the major goal of regenerative medicine-based strategies. Urodynamic evaluation is important for the validation of voiding characteristics and guidance of scaffold adjustments [21]. Physiological testing data indicated that ASCs-seeded BAMG-SF can be successfully employed for bladder augmentation, leading to an improved micturition profile. This result was accompanied by superior bladder functional recovery, as demonstrated by urodynamic studies. On the contrary, the increased collagen deposition, reduced angiogenesis and innervation hampered the function of the bladder detrusor muscles in the BAMG-SF group. The residual volume increased in both acellular and ASCs-seeded BAMG-SF groups, which was possibly associated with the augmented bladder capacity [27].

The full formation of the bladder trilayer architecture, consisting of muscle, serosa, and urothelium, with a histological appearance approaching that of normal bladder tissue provides the foundation of bladder functional restoration. ASCs accelerated angiogenesis and improved the innervation rate in the BAMG-SF-ASCs group, which led to well-organized smooth muscle regeneration and the orchestrated cooperation of detrusor smooth muscle and the nervous system. It is reasonable to speculate that angiogenesis enhances the histological regeneration of bladder wall constituents because it provides oxygen and nutrients to the regenerated tissue. The sequential process of angiogenesis includes angioblast proliferation and differentiation into endothelial cells, tube formation into a capillary network or plexus, and pericyte and smooth muscle cell recruitment, followed by the remodelling and shaping of the endothelial tube network into distinct capillary beds through processes mediated by VEGF and other angiopoietic factors [28].

The detailed mechanism of enhancing angiogenetic potential by incorporating stem cells into a scaffold remains to be elucidated. There are unclear and even inconsistent explanations of the role stem cells play in promoting angiogenesis and smooth muscle regeneration. Zambon et al. could not conclude whether Vybrant CM-DiI-labelled ASCs, muscle-derived stem cells and α-SMA^+^ smooth muscle cells are of the same origin [16]. However, in our previous study, we only detected cells positive for both CM-DiI and α-SMA, indicating that ASCs differentiated into smooth muscle cells but not CD31^+^ endothelial cells [15]. Sharma et al. concluded that human mesenchymal stem cells or CD34^+^ haematopoietic stem/progenitor cells become incorporated into the vessel wall at the onset of neovascularization, or as pericytes in nude mice [29]. While conventional stem cell therapies focus mainly on the ability of cells to differentiate and replace damaged or injured tissue, increasing evidence suggests that stem cells also exert functional activity by secreting bioactive factors that stimulate local and systemic responses [30, 31].

Our study found that the ASCs elevated the expression of SDF-1α and its receptor CXCR4, thereby contributing to VEGF-mediated angiogenesis. The role of this pathway in embryonic vasculogenesis has also been demonstrated, as SDF-1α and CXCR4 double-knockout mice exhibit severe blood vessel abnormalities [32]. Activation of the ERK 1/2 and AKT pathways is known to be involved in CXCR4-mediated angiogenesis. However, the pathway responsible for VEGF production varies among cell types [33]. SDF-1α was reported to promote glioma stem cell VEGF production and tumour angiogenesis via the phosphoinositide 3-kinase/AKT pathway, but not the mitogen-activated extracellular signal-regulated kinase/ERK pathway [34]. In contrast, SDF-1α was also reported to increase VEGF gene expression in human arterial endothelial cells through ERK 1/2 activation [35]. In this study, we speculated that the increased VEGF production after ASCs incorporation was mainly associated with the activation of ERK 1/2. One possible explanation of the enhanced VEGF production is that the ERK 1/2 downstream transcriptional factor early grow response protein 1 not only up-regulates the expression of platelet-derived growth factor and VEGFR2 but also triggers the expression of VEGF itself [36]. Further exploration is still warranted to determine whether AKT activation also involved in the observed VEGF-mediated angiogenesis and whether these two pathways cross-talk or act independently. In addition, in facilitating angiogenesis, SDF-1α/CXCR4 exerts synergistic effects with VEGF to stimulate the proliferation and tube formation of endothelial cells by mobilizing, recruiting, and homing CXCR4^+^ bone marrow-derived progenitor cells [37].

It should be noted that the maturity of neovascularization and extent of reinnervation in engineered bladder remain critical obstacles to overcome for the future clinical application of this ASCs-seeded BAMG-SF strategy. More investigations are warranted to examine the generation of a coherent and contracting bladder segment, including video cystogram, uniaxial tension test and bladder wall tension [38]. More effective ASCs delivery or proangiogenic pretreatments are warranted to achieve a further enhanced angiogenic potential, and experiments with larger sample sizes conducted using diseased bladder models or large animals are required.

## MATERIALS AND METHODS

### Scaffold preparation

After being harvested from 3-month-old pigs (Shanghai Super-B&K Laboratory Animal Co. Ltd., Shanghai, China), porcine bladder tissues were separated from the adjacent adipose tissue and facia and then rinsed in 4 °C phosphate-buffered saline (PBS; pH 7.2-7.4). The separated porcine bladder was dissected into two parts in the middle line between ureteral openings. The bladder trigone was removed, and the remaining tissues were partitioned into pieces of 5 cm × 5 cm. The serosal layer was removed with fine scissors. The submucosa was micro-dissected and isolated from the mucosal and muscular layers with micro-scissors under a microscope (M165 FC, Leica Microsystems Ltd., Heerbrugg, Germany). The lamina propria was then treated sequentially with distilled water in a stirring flask (200 rpm, 4 °C, 48 h), 0.03% trypsin (Gibco, Thermo Fisher Scientific Inc., MA, USA) for 1 h at 37 °C, 0.2% Triton X-100 and 0.1% (v/v) ammonium hydroxide for 7 days at 37 °C. The solution was refreshed every day. The resulting matrix was washed with distilled water for 2 days at 4 °C and stored in 75% ethanol. The complete elimination of cellular nuclei from the BAMG was confirmed by histological evaluation and residual DNA content quantification in our previous work [18].

The 3D porous SF layer was prepared according to previously reported procedures [20] and incorporated on the muscle layer surface of the BAMG. Briefly, *Bombyx mori* cocoons were boiled for 30 min in an aqueous solution of 0.02 M Na_2_CO_3_ and rinsed thoroughly with deionized water to remove the glue-like sericin protein. After being air-dried at room temperature, the degummed silk fibres were dissolved in 9.3 M LiBr solution for 4 h at 60 °C. This solution was dialyzed against deionized water using a cellulose dialysis bag (MWCO = 12,000, Pierce, IL, USA) for 3 days to remove LiBr. The resulting SF solution was further purified by centrifugation at 12,000 rpm for 10 min to remove impurities and aggregates formed during dialysis. The final concentration of the aqueous SF solution was determined by weighing the remaining solid after drying, and the solid was stored at 4 °C prior to experimental use. After being washed in distilled water to remove remaining alcohol, BAMG was trimmed into 15 mm × 15 mm square pieces and placed in a prepared rectangular casting vessel (bottom: 15 mm × 15 mm). SF solution (300 μl, 2% w/v) was slowly poured into the vessel. The whole material was transferred to the refrigerator and then stored for 2 days at -20 °C. The frozen composite was then lyophilized to remove the water and the other solvents to form a porous SF scaffold on a BAMG base. The composite was then sterilized by Co^60^ radiation and subjected to surgical procedures described below. All chemicals were purchased from Sigma-Aldrich Co. LLC., MO, USA.

### ASCs culture, labelling and seeding

The inguinal adipose tissues of four 2-week-old Sprague Dawley (SD) rats (Shanghai Super-B&K Laboratory Animal Co. Ltd., Shanghai, China) were isolated and rinsed with 0.25% chloromycetin three times. The tissues were cut into small pieces and digested with 0.1% type I collagenase (Sigma-Aldrich Co. LLC., MO, USA) for 1 h at 37 °C. After filtration through a 200-mm nylon filter mesh (BD Falcon, Corning Inc., NY, USA) and centrifugation (160 g, 10 min, 37 °C), the isolated cells were resuspended in Dulbecco’s modified Eagle’s medium (DMEM; Gibco, Thermo Fisher Scientific Inc., MA, USA) containing 10% foetal bovine serum (FBS; Gibco, Thermo Fisher Scientific Inc., MA, USA) and 1% penicillin-streptomycin solution (Gibco, Thermo Fisher Scientific Inc., MA, USA). The cells were seeded in two 10-cm cell culture plates (BD Falcon, Corning Inc., NY, USA) and incubated at 37 °C in an atmosphere of 5% humidified carbon dioxide. The cells were observed daily under an inverted phase-contrast microscope and were passaged with a ratio of 1:3 upon reaching 80-90% confluence. The culture medium was changed every 2 days. ASCs identification was performed as in our previous study by flow cytometry analysis for CD29, CD9, CD105 and CD45 [39], as well as differentiation into adipocytes and osteoblasts [15].

The cytocompatibility of BAMG-SF-conditioned medium with rat ASCs was evaluated by a Cell Counting Kit-8 (CCK-8) (Dojindo Laboratories, Kumamoto, Japan) according to the manufacturer’s protocol. In brief, fourth passage rat ASCs were pre-seeded at 1000 cells/well into 96-well plates (BD Falcon, Corning Inc., NY, USA) in DMEM (10% FBS) and cultured at 37 °C in 5% humidified CO_2_ for 24 hours. They were then cultured with either BAMG-SF-conditioned medium (produced by immersing BAMG-SF in DMEM at 37 °C for 48 hours) or control DMEM. At 0, 1, 4, and 7 days, culture media were aspirated and replaced by fresh DMEM; 10 μl of CCK-8 was then added. After 2 hours of incubation at 37 °C in 5% humidified CO_2_, absorbance at 450 nm was measured using a microplate reader (Varioskan, Thermo Fisher Scientific Inc., MA, USA).

Cell Tracker CM-DiI (Invitrogen, Thermo Fisher Scientific Inc., NY, USA) was used according to the manufacturer’s protocol to label the ASCs and observe their transformation *in vivo*. Briefly, third passage rat ASCs were incubated with 2 mM CM-DiI for 5 min at 37 °C, followed by a 15-min incubation at 4 °C. Incubation at the latter temperature allowed the dye to label the plasma membrane while slowing down endocytosis, thus reducing dye localization into cytoplasmic vesicles. After labelling, the cells were washed twice with PBS and resuspended in fresh DMEM containing 10% FBS. The labelled ASCs were seeded onto the BAMG-SF by placing 50 μl of the cell suspension (1×10^8^ cells/ml) onto the SF layer. Three millilitres of DMEM containing 10% FBS was slowly added 4 h after cell seeding. The medium was changed daily for 3 days.

### SEM

SEM was used to observe the interaction between the ASCs and the BAMG-SF scaffold, according to methods describe in a previous study [40]. Briefly, ASCs-seeded BAMG-SF was prefixed with 2% glutaraldehyde for 2 h at 4 °C, washed twice with PBS, and post-fixed in 1% osmic acid for 2 h at 4 °C. After two washes with distilled water, the samples were dehydrated with a gradient of ethanol and subjected to critical-point drying. The samples were then mounted, sputter-coated with gold (NeoCaster, MP-1920NCTR, JEOL, Tokyo, Japan), and examined by SEM (NeoScope, JCM-5100, JEOL, Tokyo, Japan) from cross-sectional, top, and bottom views at 20-25 kV with different magnifications.

### Mechanical properties

Twelve randomly selected BAMG-SF pieces were cut into a dog-bone shape (10 mm×40 mm). Half were seeded with ASCs as described above, and the rest received an equal volume of DMEM only. The composites were cultured in DMEM (10% FBS) for 3 days and then subjected to mechanical testing by a biomechanical analyser (Instron 5542, Illinois Tool Works Inc., IL, USA); the maximal load and elastic modulus were measured as previously described [41]. The interval length of the two grippers was set at 10 mm with a gradual moving speed of 25 mm/min until complete scaffold rupture.

### Experimental animals

The SD rats were acclimatized and housed in wire-bottomed cages with free access to food and water in temperature-controlled, pathogen-free animal room facilities (20-22 °C, humidity 40-70%, 12-h day/night cycle) for one week before the experiments. BAMG-SF was evaluated in a bladder augmentation model using 8-week-old adult male SD rats. The animals were randomly divided into three groups: the ASCs-seeded bilayer BAMG-SF group (BMAG-SF-ASCs group, n = 18), the acellular bilayer BAMG-SF group (BAMG-SF group, n = 6), and the cystotomy group (control group, n = 6). The rats in the BAMG-SF-ASCs group were sacrificed at 2, 4, and 12 weeks after bladder augmentation (n = 6 rats per time point). Meanwhile, the rats in the remaining groups were sampled at 12 weeks post-implantation.

The body weight of the rats was recorded pre- and post-operatively. The rat blood samples were subjected to routine blood testing via a haematology analyser (Genius, KT-6300, Genrui Biotech Inc., Shenzhen, China), and serum biochemistry and electrolyte measurements via a biochemical analyser (7180, Hitachi High-Technologies Corporation, Tokyo, Japan) at 2, 4, and 12 weeks post-implantation. All animal procedures were approved and supervised by the Animal Experimental Ethical Inspection of Shanghai Ninth People’s Hospital affiliated to Shanghai Jiao Tong University School of Medicine, under number HKDL [2016]149, and were performed in accordance with the guidelines of the China Act on Welfare and Management of Animals.

### Surgical procedures for bladder augmentation

**Figure 7:**
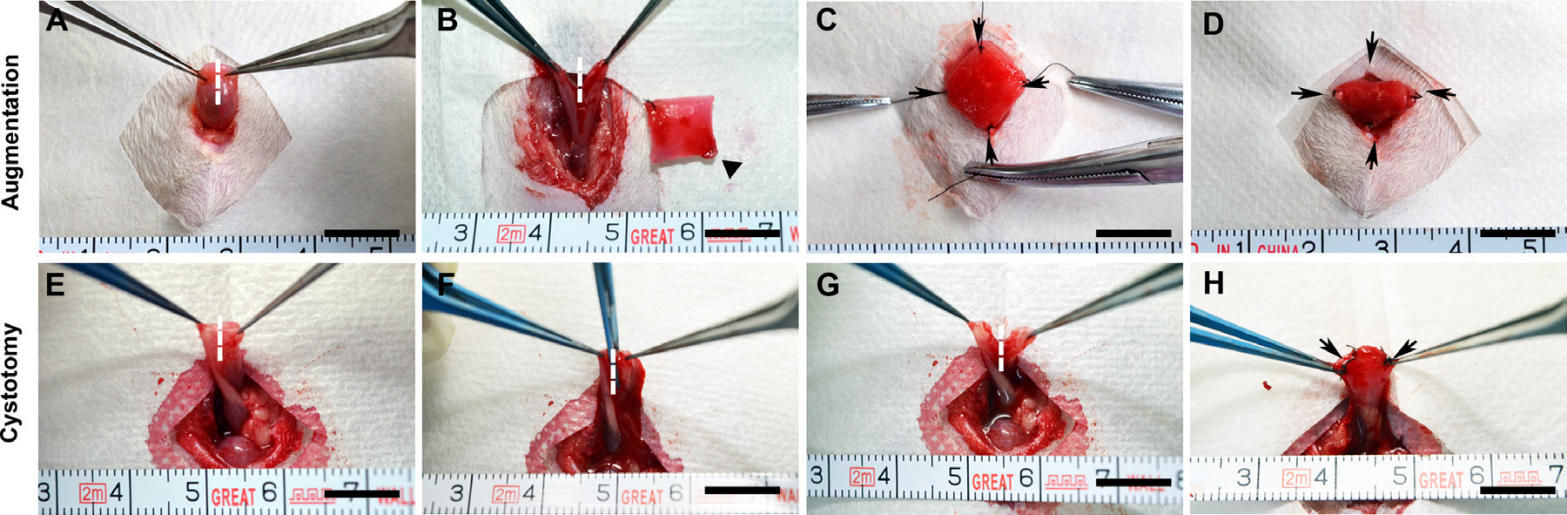
Representative photographs of the surgical procedures for bladder augmentation and cystotomy The bladders were immobilized outside the peritoneal cavity **(A, E)** and subjected to a longitudinal incision of approximately 1 cm in size in the bladder apex. **(B, F)**. In the augmentation groups, the ASCs-seeded BAMG-SF scaffold (10×10 mm) was incorporated into the bladder defect by fixation of the four corners using non-absorbable 5-0 sutures as markers of the regenerated area and then anastomosed into the bladder defect using continuous absorbable 8-0 sutures **(C, D)**. The cystotomy group only received the bladder incision, which was immediately closed using sutures **(G, H)**. Scale bar = 1 cm. Dashed lines mark the incision area. Black triangles indicate the scaffold. Black arrows note the marking sutures between the native bladder and the ASCs-seeded BAMG-SF scaffold.

After the rats were anaesthetized by an intraperitoneal injection of pentobarbital (30 mg/kg), the bladder was mobilized from the peritoneal cavity and fixed using atraumatic forceps (Figure 7A, 7E). The apex of the bladder was incised longitudinally at the midline (approximately 1 cm) (Figure 7B, 7F). In the augmentation groups, the bladder defect was marked by four non-absorbable 5-0 polypropylene sutures in each corner and anastomosed with scaffolds by absorbable 8-0 polyglactin (Ethicon, Johnson & Johnson Services, Inc., NJ, USA) in a continuous running fashion (Figure 7C, 7D). In the cystotomy group, the bladder defect was anastomosed immediately after incision (Figure 7G, 7H). A watertight seal was confirmed by filling the bladder with sterile saline via instillation through a 30-gauge hypodermic needle. Locations where repaired bladder defects leaked were reinforced with interrupted sutures of absorbable 8-0 polyglactin (Ethicon, Johnson & Johnson Services, Inc., NJ, USA).

### Conscious cystometry

The rats underwent conscious cystometry at 12 weeks post-implantation, as previously described with modifications [42]. Briefly, after administering anaesthesia with pentobarbital (30 mg/kg, intraperitoneal injection), the bladder was extruded and a slack purse-string suture was created on the dome, through the centre of which a PE-50 tube was inserted. The rest of the PE-50 tube was placed on the subcutaneous tunnel from the abdominal opening to the upper dorsum. The rats were subjected to conscious cystometry in a metabolic cage without restraint 5 days after bladder catheterization. The PE-50 tube was released and connected to a pressure transducer (Labourie Medical Technologies, Brossard, Canada) and an infusion pump (infusion speed: 100 μl/min) via a 3-way stopcock. The voiding volume and intravesical pressure curve were recorded spontaneously when the pressure curve became stable. At least 3 micturition cycles were recorded for the cystometrogram, drawn by Origin 9.0 (Origin Lab, Northampton, MA, USA), and the urodynamic parameter analysis, including residual volume (subtracting the voiding volume by the infused saline volume), voiding volume, bladder capacity (the infused volume during one micturition cycle), basal pressure (the pressure just after the end of micturition), threshold pressure (the pressure immediately before the initiation of micturition), peak micturition pressure (the maximum pressure during micturition), bladder compliance (calculated by acquiring the ratio of the volume instilled during the filling phase and the change in pressure, compliance = ΔV/ΔP) [43].

### Gross bladder morphology and retrograde cystography

At 2, 4 and 12 weeks post-implantation, the rat bladders were injected with contrast medium (30% iopamidol, GE Healthcare, IL, USA) by intravesical instillation while under general anaesthesia (pentobarbital, 30 mg/kg, intraperitoneal injection) until the first urethral urine leakage. An X-ray film was obtained for each experimental subject. Then, the bladder was extruded, and its gross morphology was examined *in vivo*.

### Histological and immunofluorescence analysis

After the rats were euthanized by CO_2_ asphyxiation, the rat bladders were excised for standard histological processing [29]. After ligating the bladder outlet by two non-absorbable 5-0 polypropylene sutures, the bladder was harvested by cutting between the two sutures. The bladder samples and aforementioned CM-DiI-labelled ASCs-seeded BAMG-SF composites were immediately fixed in 4% formaldehyde overnight at 4 °C. Then, the samples were dehydrated through a series of graded ethanol solutions and embedded in paraffin. The paraffin-embedded tissue samples were sectioned onto glass slides, and the slides were deparaffinized for 30 mins at 60 °C followed by treatment with xylene, graded ethanol and double-distilled water. The bladder sections were subjected to H&E stain, MTS, and immunohistochemical stain for MPO (1:50 dilution, ab9535, Abcam, Cambridge, MA, USA) and CD68 (1:150 dilution, ab31630, Abcam, Cambridge, MA, USA). The ASCs-seeded BAMG-SF sections underwent H&E stain and DAPI counterstaining separately.

The immunofluorescence analysis was performed to detect the urothelium-associated protein cytokeratin (CK, AE1/AE3), the contractile smooth muscle marker α-SMA, and the endothelial and neuronal markers CD31 and NeuN using the following primary antibodies: anti-cytokeratin AE1/AE3 (1:50 dilution, ab1747070, Abcam, Cambridge, MA, USA), anti-α-SMA (1:150 dilution, ab32575, Abcam, Cambridge, MA, USA), anti-CD31 (1:100 dilution, ab119339, Abcam, Cambridge, MA, USA), and anti-NeuN (1:150 dilution, ab177487, Abcam, Cambridge, MA, USA). The sections were then incubated with species-matched fluorescein isothiocyanate (FITC)-conjugated secondary antibodies (Millipore, Billerica, MA, USA), and nuclei were counterstained with DAPI.

Specimens were visualized using a Nikon Eclipse 80i fluorescence microscope (Nikon Instruments Inc., Tokyo, Japan), and representative images were acquired using NIS-Elements 4.0 (Nikon Instruments Inc., Tokyo, Japan). The numbers of neutrophils (MPO^+^) and macrophages (CD68^+^), the percentages of positive protein expression corresponding to SF and collagen per total area of regenerated area, and the vessel densities and diameters were calculated from 6 randomly selected fields of view of three slides using ImageJ 1.50i (National Institutes of Health, Bethesda, MD, USA).

### Western blot analysis

The proteins in the bladder samples were harvested by radioimmunoprecipitation assay lysis buffer, quantified by a bicinchoninic acid Protein Assay Kit (Beyotime Biotechnology, Shanghai, China), and separated by 10% sodium dodecyl sulphate polyacrylamide gel electrophoresis. Then, the samples were subjected to immunoblotting with antibodies to SDF-1α (1:500 dilution, ENT4225, Elabscience Biotechnology Co., Ltd., Wuhan, China), CXCR4 (1:100 dilution, ab124824, Abcam, Cambridge, MA, USA), VEGF (1:1000 dilution, ab32152, Abcam, Cambridge, MA, USA), VEGFR2 (1:300 dilution, bs-10412R, Biosynthesis Biotechnology Co. Ltd., Beijing, China), AKT (1:1000 dilution, AF6261, Affinity Biosciences Inc., OH, USA), phosphorylated AKT (1:1000 dilution, AF0016, Affinity Biosciences Inc., OH, USA), ERK 1/2 (1:1000 dilution, AF0155, Affinity Biosciences Inc., OH, USA), and phosphorylated ERK 1/2 (1:1000 dilution, AF1014, Affinity Biosciences Inc., OH, USA), and were then transferred onto polyvinylidene fluoride membranes (Millipore, Darmstadt, Germany). The membranes were blocked in 5% non-fat milk at room temperature for 2 h and incubated with primary antibodies overnight at 4 °C, followed by incubation with horseradish peroxidase-conjugated secondary antibodies (1:50000 dilution, BA1054, Boster Biological Technology Co. Ltd., Wuhan, China) for 2 h at room temperature. The protein bands were visualized using an enhanced chemiluminescence detection kit (NCI5079, Thermo Fisher Scientific Inc., MA, USA) on X-ray film (XBT-1, Eastman Kodak Company, NY, USA). β-Actin (1:200 dilution, BM0627, Boster Biological Technology Co. Ltd., Wuhan, China) was used as an internal control.

### RNA isolation & qRT-PCR

Total RNA was extracted from the regenerated bladder tissue using TRIzol reagent (Invitrogen, Thermo Fisher Scientific Inc., NY, USA). cDNA was synthesized by reverse transcription of total RNA using the Hiscript Reverse Transcriptase (Vazyme Biotech Co. Ltd., Nanjing, China), according to the manufacturer’s instructions. cDNA products were diluted 10 times, 4 μl was used as the templates for qRT-PCR. The transcription levels of the genes for VEGF, VEGFR2, SDF-1α, and CXCR4 were determined by qRT-PCR analysis with an Eco™ Real-Time PCR System (ABI7900, Illumina, Inc., CA, USA) using Power SYBR Green PCR master mix (2×) (Vazyme Biotech Co. Ltd., Nanjing, China). qRT-PCR was performed with a protocol of 50 °C for 2 min, 95 °C for 10 min, 40 cycles at 95 °C for 30 secs and 60 °C for 30 sec. Target mRNA expression levels were normalized to the housekeeping genes of rat β-actin as an internal control for quantification by 2^-(∆∆Ct)^. The primers used for the qRT-PCR analysis are listed in Table 3. Each assay was performed in triplicate.

**Table 3:** Oligonucleotide sequences of qRT-RCR primers

mRNA	Sense strand (5′-3′)	Antisense strand (5′-3′)
SDF-1α	ATGCCCCTGCCGATTCTTTG	TTGTTGCTTTTCAGCCTTGC
CXCR4	CGGTCATCCTTATCCTGGCT	CTCTTGAATTTGGCCCCGAG
VEGF	CGTCTACCAGCGCAGCTATTG	CTCCAGGGCTTCATCATTGC
VEGFR2	CTTCATAATAGAAGGCGTCCAG	ATAAGGCAAGCGTTCACAGC
β-Actin	CACGATGGAGGGGCCGGACTCATC	TAAAGACCTCTATGCCAACACAGT

### Statistical analysis

All data are expressed as the mean ± standard deviation. Statistical analyses were performed using a two-tailed Student’s t-test or one-way analysis of variance (ANOVA) with the Bonferroni post hoc test in GraphPad Prism 5.01 (GraphPad Software Inc., San Diego, CA, USA). Two-sided P<0.05 was considered statistically significant.

## Abbreviations

ASCs: adipose-derived stem cells
BAMG: bladder acellular matrix graft
SF: silk fibroin
H&E: haematoxylin-eosin
MTS: Masson’s trichrome stain
SDF-1α: stromal-derived factor 1α
CXCR4: CXC chemokine receptor 4
ERK 1/2: extracellular signal-regulated kinases 1/2
AKT: protein kinase B
VEGF: vascular endothelium grow factor
VEGFR2: VEGF receptor 2
SD: Sprague Dawley
MPO: myeloperoxidase
CK: cytokeratin
α-SMA: α-smooth muscle actin
